# Estimation of the work of adhesion between ITO and polymer substrates: a surface thermodynamics approach

**DOI:** 10.55730/1300-0527.3518

**Published:** 2022-11-22

**Authors:** Salih ÖZBAY

**Affiliations:** Department of Chemical Engineering, Faculty of Engineering and Natural Sciences, Sivas University of Science and Technology, Sivas, Turkey

**Keywords:** Indium tin oxide, work of adhesion, surface free energy, contact angle, polymer substrate

## Abstract

Indium tin oxide (ITO) is one of the most widely used semiconductor among transparent conducting oxides (TCOs) due to their electrical conductivity and optical transparency properties. Since the development of low temperature deposition methods, coating of ITO on polymer substrates especially for use in flexible electronics has been a popular topic. The existence of adequate adhesion strength between ITO and polymer is critical in producing a successful film. Nowadays, polycarbonate (PC), poly(methyl methacrylate) (PMMA) and polyethyleneterephtalate (PET) are frequently used as substrates for such coatings. However, there may be other polymeric alternatives that have a potential to be used for this purpose in the future. To evaluate these alternatives, work of adhesion (*W_a_*) knowledge between ITO and polymers is necessary, and it has not been handled systematically previously. In this study, the interphase interaction parameters and *W_a_* values between ITO and various polymers were calculated based on the Dupré, Fowkes and Girifalco-Good equations. PC, PMMA, PET, polystyrene (PS), polyphenylene sulfide (PPS), Nylon 66, polypropylene (PP), polyvinylchloride (PVC), styrene-butadiene rubber (SBR), high density polyethylene (HDPE), low density polyethylene (LDPE), polyvinyl acetate (PVAc), polyvinyl fluoride (PVF), polyvinylidene fluoride (PVDF), polytetrafluoroethylene (PTFE), polytrifluoroethylene (PTrFE) and polyperfluoroalkylethyl acrylate (PPFA) were considered as substrate material. Surface free energy (SFE) components calculated by acid-base, geometric mean and harmonic mean approaches for polymeric substrates were used during the calculations. In the present study, the polymers that can be used as substrates were evaluated in terms of adhesion ability to ITO, the significance of calculation methods on *W_a_* values were also investigated simultaneously. It was determined that the *W_a_* between ITO and polymer substrates was directly related with the total SFE value of the polymers.

## 1. Introduction

Sn doped In_2_O_3_, also known as indium tin oxide (ITO), is one of the most widely used n-type semiconductor among transparent conducting oxides (TCOs) due to their electrical conductivity and optical transparency. It is typically comprised from the solution of 90% In_2_O_3_ and 10% SnO_2_ by weight, and used in many technological systems such as liquid crystal displays (LCDs), organic light-emitting diodes (OLEDs), photovoltaics and biosensors [1,2]. The demand to produce ITO films by adjusting the electrical conductivity and optical transparency has led to the development of many deposition methods [3]. DC magnetron sputtering [4], RF magnetron sputtering [5], ion beam sputtering [6], electron beam evaporation [7], chemical vapor deposition [8] and chemical solution deposition [9] are the examples of deposition methods used to coat ITO on a suitable substrate. The use of glass as substrate is quite common in the field due to its ability to withstand high temperatures, since a significant part of the coatings made with the aforementioned methods was carried out by heating the substrate to elevated temperatures of over 200 °C [10].

However, the use of polymeric materials as substrates to produce ITO films is critical for many applications such as plastic LCD devices, electromagnetic interference shielding materials and flexible electronics [11–14]. Since the development of low temperature deposition methods [3,10,15–19], preparation of ITO films by using appropriate polymer substrates has been a popular topic. Polycarbonate (PC) [3,14,20,21], polyethyleneterephtalate (PET) [17,20,22–26] and poly(methyl methacrylate) (PMMA) [5,26] are the most commonly used polymer substrates for this purpose. Although there are various advantages of using polymers as substrate, insufficient adhesion of ITO to the polymeric substrates is still a big problem. In order to overcome this, many modification methods have been applied to the polymer substrate or ITO. Air plasma, argon plasma and O_2_ plasma are some of the plasma treatment methods used for this purpose. During these processes, surface free energy (SFE) properties of ITO and substrate have been put forward as an important parameter many times [4,21], because a detailed SFE knowledge for solid surfaces is critical in evaluating of many interface phenomena such as adhesion, adsorption, wettability, and lubrication behaviour. For example, Vunnam et al. improved the ITO surface by changing the wettability properties of ITO for direct writing of silver nanoparticulate ink micropatterns by using air plasma treatment [27]. Lee et al. increased the SFE of the ITO by using argon atmospheric pressure plasma, and reported that the optoelectronic properties of the ITO can be optimized by this way [28]. You and Dong treated the surface of ITO by O_2_ plasma to improve the ITO/polymer interface for use in organic light emitting diodes [29]. Recently, we adjusted SFE properties of the PC substrate by O_2_ plasma treatment to prepare ITO-based transparent and conducting multilayer thin films that could be potentially used in optoelectronic industry [21]. In addition to plasma treatment methods, chemical treatments were also applied in order to modify SFE properties of TCOs. For example, Arazna et al. reported that the treatment of ITO surface in ultrasonic bath using organic solvents such as acetone, ethyl alcohol and isopropyl alcohol is effective for increasing the SFE of the ITO [30]. Davenas et al. immersed the ITO substrate in a solution of 2-chloroethylphosphonic acid in order to functionalize it with a molecular layer, and increased the SFE value of the ITO for improvement of charge injection in organic light emitting diodes [31]. Similarly, Besbes et al. increased the SFE value of ITO with 2-chloroethanephosphonic acid and developed an ITO/polymer interface that is more suitable for use in organic light emitting devices by this way [32]. Apart from these studies, silane based chemicals are effective for improving the adhesion ability of ITO’s contact surface due to the Si-O bonds that can be react with the hydroxyl groups of ITO [2,33–35]. In this context, Chiang and Hsieh used five different types of organo-functional silanes containing vinyl, epoxy, amino, methacrylic and acrylic groups in the cationic polymerization of epoxide resin to enhance adhesion between epoxide resin and ITO [36]. Maksimenko et al. used 3-methacryloxypropyltrimethoxysilane to improve the attachment between ITO and polyvinylpyrrolidone for the synthesis of ITO/polymer nanocomposites [37]. Similarly, Ginzburg-Turgeman et al. reported that the use of 3-(trimethoxysilyl)propyl methacrylate as a silane-based molecular adhesive was very effective in establishing covalent attachment between PMMA and ITO, and thus improved the adhesion [38]. All of these studies clearly indicate that the interfacial tension forces, and hence work of adhesion (*W_a_*), between ITO and organic substrates have an important role in order to design a desired structure.

Besides, there are many unknown information in this field about the adhesion relationships between ITO and polymer-based substrates. As known, there are numerous methods in the literature to calculate SFE from contact angle results. Which of these might be better associated with adhesion of ITO to polymer substrates? On the other hand, although PC, PMMA and PET are most commonly used substrates for ITO-based coatings, what other industrial polymers would be a good candidate as substrates for such coatings? In order to answer these questions, the adhesion between ITO and various polymer substrates must be known. In this context, calculation of thermodynamic work of adhesion between interlayers by using SFE values stands out as a good alternative [4].

The main aim of this study is to estimate the thermodynamic *W_a_* values between ITO and various common polymeric substrates as accurately as possible by using previously reported contact angle and SFE values of ITO and polymeric substrates. To do this, SFE components of ITO and polymeric substrates have been listed by using acid-base [39], geometric mean [40] and harmonic mean [41] approaches. Common and industrial polymers such as polystyrene (PS), poly(methyl methacrylate) (PMMA), polycarbonate (PC), polyethyleneterephtalate (PET), Nylon 66, polypropylene (PP), polyvinylchloride (PVC), styrene-butadiene rubber (SBR), polyphenylene sulfide (PPS), high density polyethylene (HDPE), low density polyethylene (LDPE), polyvinyl acetate (PVAc), polyvinyl fluoride (PVF), polyvinylidene fluoride (PVDF), polytetrafluoroethylene (PTFE), polytrifluoroethylene (PTrFE) and polyperfluoroalkylethyl acrylate (PPFA) were selected as substrate material. Following this, the interaction parameter of interphase (*ϕ*) and *W_a_* values between ITO and selected polymeric substrates were estimated using the SFE values based on the Dupré [42], Fowkes [43–45] and Girifalco-Good equations [46]. Finally, alternative polymers to be used as substrates for ITO coatings have been proposed considering calculated *W_a_* values.

## 2. Theoretical background and estimation of the work of adhesion between ITO and polymer substrates

Thermodynamic work of adhesion ( $W_{12}^{a}=-\Delta G_{12}^{a}$) between two material surfaces can be defined as the reversible work required to separate two phases at the interface to infinite distance [47,48]. According to Dupré equation, work of adhesion between two phases depending on the interfacial tensions can be written as [42]


(1) $$-\Delta G_{12}^{a}=W_{12}^{a}=\gamma_{1}+\gamma_{2}-\gamma_{12}.$$

*W_a_* can also be described by the sum of intermolecular interactions as proposed by Fowkes [43–45],


(2) $$W_{a}=W_{a}^{d}+W_{a}^{h}+W_{a}^{p}+W_{a}^{i}+W_{a}^{\pi }+W_{a}^{da}+W_{a}^{e}+\cdots ,$$

where superscript *d* denotes London-dispersion forces, *h* denotes hydrogen-bonding, *p* denotes dipole-dipole (polar) interactions, *i* denotes dipole-induced dipole interactions, *π* denotes π-bonds, *da* denotes donor-acceptor bonds, and *e* denotes electrostatic interactions. Fowkes suggests that London-dispersion forces are always present, and $W_{a}^{d}$ component is usually dominant when compared to the other components [44].

According to Berthelot’s approach, attractive constants between like (*A*_aa_ and *A*_bb_) and unlike (*A*_ab_) molecules can be expressed as [46,49]


(3) $$A_{ab}=\sqrt{A_{aa}A_{bb}}.$$

Girifalco and Good modified Equation (3) by using the free energies of adhesion and cohesion for two phases. Their basic principle is to equate $A_{ab}/\sqrt{A_{aa}A_{bb}}$ ratio to the interphase interaction parameter (*ϕ*) [46]. According to Girifalco and Good, the interphase interaction parameter can be defined as,


(4) $$\varphi =-\frac{\Delta F_{ab}^{a}}{\sqrt{\Delta F_{a}^{c}\Delta F_{b}^{c}}},$$

where $\Delta F_{ab}^{a}$ denotes the free energy of adhesion for the interface between *a* and *b* phases, $\Delta F_{a}^{c}$ and $\Delta F_{b}^{c}$ denote free energy of cohesion for *a* and *b* phases, respectively. This equation can also be defined as [50],


(5) $$\varphi =\frac{W_{a}}{\sqrt{W_{c1}W_{c2}}},$$

where *W_c_* denotes work of cohesion, and it can be expressed using surface tension terms as.


(6) $$W_{c}=2\gamma .$$

By combining Equations (1), (5), and (6), interfacial tension between two material surfaces can be written as.


(7) $$\gamma_{12}=\gamma_{1}+\gamma_{2}-2\varphi \sqrt{\gamma_{1}\gamma_{2}}.$$

By combining Equations (1) and (7), the main equation of Girifalco-Good for *W_a_* between two material surfaces can be expressed as.


(8) $$W_{a}=2\varphi \sqrt{\gamma_{1}\gamma_{2}}.$$

Equation (8) can be written for polymer/ITO systems as


(9) $$W_{a}=2\varphi \sqrt{\gamma_{P}\gamma_{ITO}},$$

where *γ_P_* is SFE of the polymer substrate, and *γ_ITO_* is SFE of the ITO. The *ϕ* value of the interphase between polymer and ITO can be written by geometric mean approach as [4,40,50],


(10) $$\varphi =\sqrt{X_{P}^{d}X_{ITO}^{d}}+\sqrt{X_{P}^{p}X_{ITO}^{p}},$$

where $X_{j}^{d}$ denotes dispersive component fraction of the SFE, $X_{j}^{p}$ denotes polar component fraction of the SFE, subscript *j* represents the polymer or ITO, and $X_{j}^{d}+X_{j}^{p}=1$.

The *ϕ* value of the interphase between polymer and ITO can be written by harmonic mean approach as [4,41,50].


(11) $$\varphi =2\;\left(\frac{X_{P}^{d}X_{ITO}^{d}}{g_{P}X_{P}^{d}+g_{ITO}X_{ITO}^{d}}+\frac{X_{P}^{p}X_{ITO}^{p}}{g_{P}X_{P}^{p}+g_{ITO}X_{ITO}^{p}}\right).$$

The parameters in Equations (10) and (11) are calculated as


(12) $$X_{j}^{d}=\frac{\gamma_{j}^{d}}{\gamma_{j}}$$

(13) $$X_{j}^{p}=\frac{\gamma_{j}^{p}}{\gamma_{j}}$$

(14) $$g_{j1}=\sqrt{\gamma_{j1}/\gamma_{j2}}.$$

Apart from *ϕ* values, the SFE values of the polymer and ITO must also be known in order to calculate *W_a_* by using Equation (9). There are three main approaches that can be used to calculate the SFE. One of them is the van Oss-Chaudhury-Good method, which is based on the acid-base (AB) approach [39]. The main equation of the van Oss-Chaudhury-Good method can be written as,


(15) $$\gamma_{LV}\;(1+\text{cos}\;\theta )=2\;\left(\sqrt{\gamma_{S}^{LW}\;\gamma_{L}^{LW}}+\sqrt{\gamma_{S}^{+}\;\gamma_{L}^{-}}+\sqrt{\gamma_{S}^{-}\;\gamma_{L}^{+}}\right),$$

where subscript S denotes solid; L liquid; V vapor, *γ^LW^* is the Lifshitz-van der Waals SFE term, and *θ* denotes contact angle. Other equations used in this method are


(16) $$\gamma_{i}^{AB}=2\;\sqrt{\gamma_{i}^{+}\;\gamma_{i}^{-}}$$

(17) $$\gamma_{i}^{Tot}=\gamma_{i}^{LW}+\gamma_{i}^{AB},$$

where subscript *i* denotes liquid or solid, $\gamma_{i}^{+}$ denotes Lewis acid parameter, $\gamma_{i}^{-}$ denotes Lewis base parameter and $\gamma_{i}^{AB}$ comprises all the electron acceptor-donor interactions. The Owens and Wendt’s method based on the geometric mean (GM) approach [40], and Wu’s method based on the harmonic mean (HM) approach [41] are the other commonly used methods for calculation of SFE and its parameters. The equations used for the determination of SFE based on the geometric mean approach and the harmonic mean approach can be written as follows, respectively:


(18) $$\gamma_{LV}\;(1+\text{cos}\;\theta )=2\;\left(\sqrt{\gamma_{S}^{d}\;\gamma_{L}^{d}}+\sqrt{\gamma_{S}^{p}\;\gamma_{L}^{p}}\right)$$

(19) $$\gamma_{LV}\;(1+\text{cos}\;\theta )=4\;\left(\frac{\gamma_{S}^{d}\;\gamma_{L}^{d}}{\gamma_{S}^{d}+\gamma_{L}^{d}}+\frac{\gamma_{S}^{d}\gamma_{L}^{p}}{\gamma_{S}^{p}+\gamma_{L}^{p}}\right),$$

where *γ^d^* denotes dispersive component of the surface tension, and *γ^p^* denotes polar component of the surface tension. The surface tension components of the test liquids used in the equations can be easily retrieved from the literature [4,21,47,51]. In brief, after determination of the *ϕ* values, SFE values calculated with AB, GM and HM approaches are used in equation (9) to determine *W_a_* values of polymer/ITO interfaces.

## 3. Results and discussion

The SFE components of ITO coated on different substrates taken from the literature and are presented in Table 1. Although used substrates have quite different physical and chemical properties, the SFE values of ITO were distributed in a narrow range. For example, the $\gamma_{S}^{Tot}$ values of ITO coated on PET and glass substrates are reported as 29.09 and 29.30 mJ/m^2^, respectively, by using acid-base approach [23,31]. Similarly, the $\gamma_{S}^{Tot}$ value of ITO coated on O_2_ plasma treated PC is reported as 32.08 mJ/m^2^ by using acid-base approach, and this value is close to $\gamma_{S}^{Tot}$ value (33.31 mJ/m^2^) of ITO deposited on a gold interlayer [21]. While the $\gamma_{S}^{+}$ parameters were reported as zero or very close to zero under all deposition conditions, the mean value for $\gamma_{S}^{-}$ was calculated as 4.38 mJ/m^2^, signifying that all of the ITO surfaces have a monopolar basic character (Table 1). As known, acid-base approach sometimes gives negative values in the square roots of $\gamma_{S}^{+}$ and $\gamma_{S}^{-}$, causing $\gamma_{S}^{AB}$ to be calculated as zero. The values close to zero originating from the negative values of the square roots of $\gamma_{S}^{+}$ caused $\gamma_{S}^{AB}$ values to be calculated as zero for ITO surfaces. For this reason, determination of the polar interactions for ITO surfaces by acid-base approach is very difficult [4,21,23].

The dispersive ( $\gamma_{S}^{d}$) and polar ( $\gamma_{S}^{p}$) components of the SFE for ITO calculated with geometric and harmonic mean approaches are listed in Table 1. The mean values for $\gamma_{S}^{d},\gamma_{S}^{p}$, and $\gamma_{S}^{Tot}$ were calculated as 28.64, 1.00, and 29.64 mJ/m^2^, respectively, by using geometric mean approach. These values are close to those obtained from acid-base approach. However, when using harmonic mean approach, the mean values for $\gamma_{S}^{d},\gamma_{S}^{p}$, and $\gamma_{S}^{Tot}$ were calculated as 30.60, 1.92, and 32.52 mJ/m^2^. These results show that SFE components calculated by harmonic mean approach are higher than the geometric mean and acid-base approaches as seen in Wu’s previous determinations [41,50]. In summary, while SFE values of ITO are distributed in a narrow range, polymers show a wide range of SFE distribution (around 7–45 mJ/m^2^) depending on their molecular structure [51]. Accordingly, the adhesion strength of ITO to polymer base substrates varies predominantly according to the SFE properties of used polymer.

SFE components of polymer to be used for substrate material must also be known in order to determine *W_a_* between ITO and the polymer. In this context, contact angle measurements, which quantifies liquid/solid interactions, are one of the most popular techniques used to determine the SFE of a surface. However, SFE components of a surface can vary depending on the calculation methods and type of liquid used. For this reason, the SFE values obtained by using the same liquids in the same calculation method can only be compared with each other. In this work, we have retrieved contact angle results of water (W), formamide (FA) and diiodomethane (DM) liquids on common and industrial polymers from previous literature reports [21,51–60], and presented in Table 2. We then listed the SFE components calculated for polymer surfaces with acid-base, geometric mean and harmonic mean approaches using W, FA and DM contact angle results (Table 3). The results show that surface wettability properties of the polymers are very different from each other. For instance, W contact angle results of the listed polymers ranged between 56° and 125°. Similar wide range distributions are also seen for FA and DM contact angle results. The wide distributions observed in contact angle results of the polymer surfaces naturally resulted in wide distributions of the SFE results. As can be seen from Table 3, $\gamma_{S}^{Tot}$ values of the listed polymers change between 7.45 mJ/m^2^ and 64.73 mJ/m^2^ depending on the chemical structure of the polymer and calculation method. In means of $\gamma_{S}^{+}$ and $\gamma_{S}^{-}$ values, most of the polymers (except PPS and PPFA) evaluated in this work have larger $\gamma_{S}^{-}$ values compared with the $\gamma_{S}^{+}$ components, indicating that most of the common polymers are on the monopolar basic character. For example, while the $\gamma_{S}^{-}$ values for PMMA, PET and PC are 15.58, 6.42 and 5.70, respectively, it is observed that $\gamma_{S}^{+}$ values for these polymers are 0 mJ/m^2^. Also, SFE values calculated by acid-base approach are close to those obtained from geometric mean approach. However, when using harmonic mean approach, SFE parameters of the polymers especially for $\gamma_{S}^{p}$ and $\gamma_{S}^{Tot}$ are found to be higher than that of acid-base and geometric mean approaches. Although all of these results help to understand the adhesion behaviour of polymers, thermodynamic *W_a_* values of ITO/polymer interlayers should be determined in order to comment on how these changes of SFE components affect the adhesion strength between ITO and polymers properly.

The interphase interaction parameter (*ϕ*), also known as Girifalco-Good interaction parameter, is a good indicator to determine the degree of interaction between two phases, and thus work of adhesion [46,61]. The *ϕ* values of ITO/polymer systems were calculated from SFE components of the polymeric substrates and ITO by applying geometric and harmonic mean approaches as explained in the theoretical background section. As seen in Table 4, all of the *ϕ* values between polymeric surfaces and ITO were close to 1 in most cases, although they varied somewhat depending on the calculation methods. This indicates that *W_a_* values are directly related to the total SFE value of the polymeric surfaces due to the nature of the Girifalco-Good calculation approach. These results also indicate that the polymers considered in this study may be good substrate candidates for ITO coatings, given that the *ϕ* values can range between 0.5 and 1.15 [46,61]. However, *W_a_* knowledge between ITO and polymers is also necessary in order to predict the extent of adhesion of ITO/polymer interfaces.

**Figure 1:**
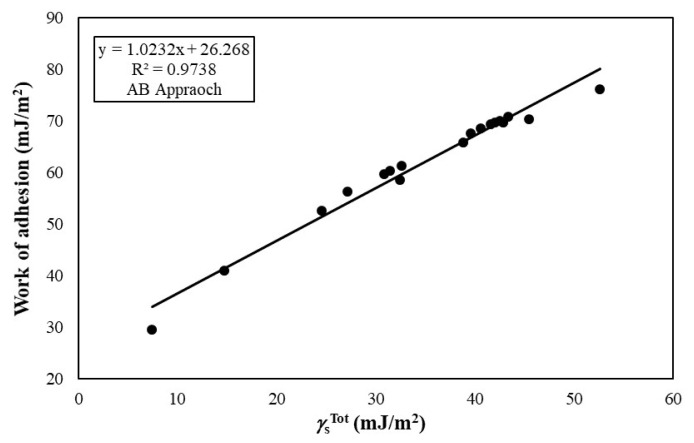
Change of work of adhesion between ITO and polymer substrates with the change of SFE values of polymer substrates calculated by acid-base approach.

**Figure 2:**
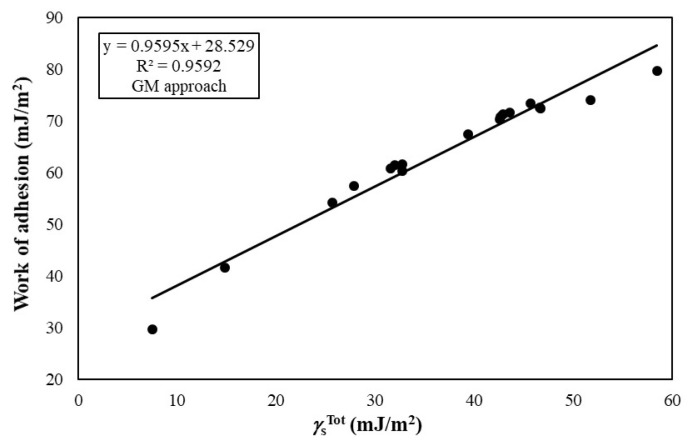
Change of work of adhesion between ITO and polymer substrates with the change of SFE values of polymer substrates calculated by geometric mean approach.

**Figure 3:**
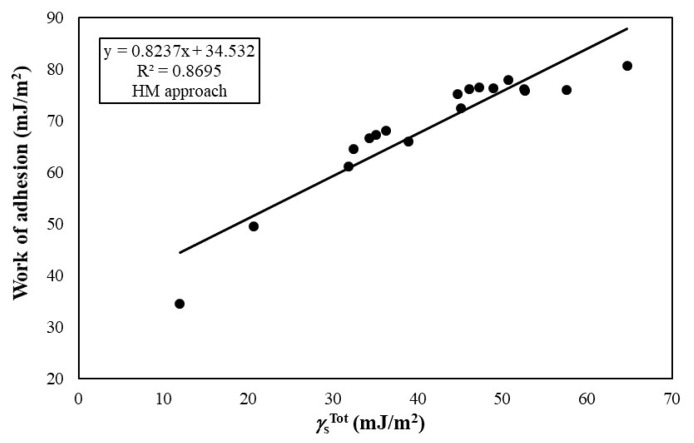
Change of work of adhesion between ITO and polymer substrates with the change of SFE values of polymer substrates calculated by harmonic mean approach.

The *W_a_* values between ITO and polymeric substrates are given in Table 4. The *W_a_* values of the ITO films to the polymeric substrates varied from 29.54 to 80.62 mJ/m^2^ depending on the type of polymer and calculation model. The *W_a_* values increased from 29.54 to 76.20 mJ/m^2^ with the increase of the $\gamma_{S}^{Tot}$ of polymeric substrates from 7.45 to 52.60 mJ/m^2^, which calculated by acid-base approach as seen in Figure 1. The regression coefficient value of the line of this graph was found to be 0.97, and at first glance, it can be thought that the use of $\gamma_{S}^{Tot}$ values determined by the acid-base approach is directly related to the calculated *W_a_* values. However, the $\gamma_{S}^{+}$ and $\gamma_{S}^{-}$ values of many surfaces reported in this work had to be considered zero due to the negative values of $\sqrt{\gamma_{S}^{+}}$ and $\sqrt{\gamma_{S}^{-}}$ [4,21,51]. This assumption caused $\gamma_{S}^{AB}$ to be calculated as zero, and contribution to the $\gamma_{S}^{Tot}$ was mainly originated from the $\gamma_{S}^{LW}$. Thus, the relationship between $\gamma_{S}^{Tot}$ and *W_a_* for ITO/polymer systems should also be evaluated by other SFE calculation approaches to verify mentioned strong correlation. According to the $\gamma_{S}^{Tot}$ values calculated from geometric mean approach, the *W_a_* values increased from 29.74 to 79.72 mJ/m^2^ with the increase of the $\gamma_{S}^{Tot}$ of polymeric substrates from 7.46 to 58.44 mJ/m^2^ as seen in Figure 2. Similar to acid-base and geometric mean approaches, the *W_a_* values calculated by harmonic mean approach were also increased sharply from 34.51 to 80.62 mJ/m^2^ with the increase of the $\gamma_{S}^{Tot}$ of polymeric substrates as seen in Figure 3. All of these results clearly show that the *W_a_* between ITO and polymeric substrates is highly correlated with the $\gamma_{S}^{Tot}$ value of the used polymeric material.

**Figure 4:**
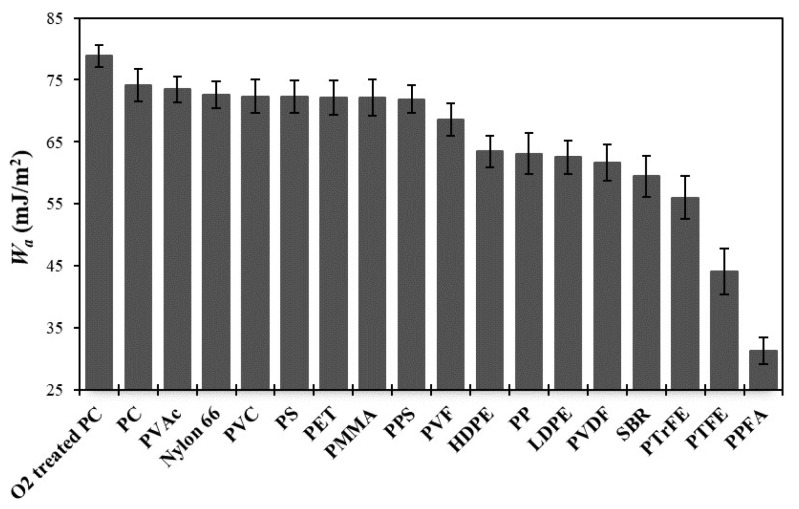
Comparison of work of adhesion between ITO and various polymer substrates by averaging the results obtained from acid-base, geometric mean and harmonic mean approaches.

In order to make a more precise comparison, the *W_a_* values between ITO and polymeric substrates were interpreted by averaging the results obtained from acid-base, geometric mean and harmonic mean approaches (Figure 4). According to the results obtained, the *W_a_* value of ITO film to O_2_ treated PC was 78.85 ± 1.8 mJ/m^2^, whereas the *W_a_* for untreated PC was calculated as 74.10 ± 2.6 mJ/m^2^. This is due to the differences in the SFE components of both surfaces. For example, the $\gamma_{S}^{Tot}$ values of O_2_ treated PC surface were 52.60, 58.44, and 64.73 mJ/m^2^ by acid-base, geometric mean and harmonic mean methods, respectively. Where as, the $\gamma_{S}^{Tot}$ values of untreated PC surface were 43.37, 45.63, and 50.68 mJ/m^2^ by acid-base, geometric mean and harmonic mean methods, respectively [21]. On the other hand, electron donor functional component ( $\gamma_{S}^{-}$) of the SFE shows the hydrogen bonding ability of the carbonyl groups present on the surface [55]. The $\gamma_{S}^{-}$ value of the PC surface increased from 5.70 to 17.54 mJ/m^2^ with O_2_ plasma treatment, and it shows that the intensity of polar oxygenated groups on the surface increases and carbonyl groups ready to form hydrogen bonds cover most of the PC surface [21]. This change may have caused higher *W_a_* at the O_2_ treated PC/ITO interface comparing to the untreated PC/ITO.

PET is another important substrate that often used for ITO coatings, and the *W_a_* value of PET/ITO interface was calculated as 72.17 ± 2.8 mJ/m^2^. This value is very close to those obtained from PS (72.27 ± 2.6 mJ/m^2^), PMMA (72.15 ± 3.0 mJ/m^2^), and PPS (71.90 ± 2.2 mJ/m^2^). Considering the very close *W_a_* values of PET and PMMA, it can be better understood why these two polymers seem to be good alternatives to each other. On the other hand, close *W_a_* values of PC, PS, PET and PPS are due to the close values of $\gamma_{S}^{LW}$ resulting from the presence of benzene rings in their structures [21,51,53]. Comparing PC, PMMA and PET, the *W_a_* values between polymer and ITO decreased in the following order: PC (74.10 ± 2.6 mJ/m^2^) > PET (72.17 ± 2.8 mJ/m^2^) > PMMA (72.15 ± 3.0 mJ/m^2^). Apart from these, PVAc (73.49 ± 2.1 mJ/m^2^), Nylon 66 (72.63 ± 2.1 mJ/m^2^), and PVC (72.35 ± 2.7 mJ/m^2^) have also high *W_a_* values at the polymer/ITO interface. This is an expected situation, because it is a known fact that PVAc is used in the adhesive industry due to its high surface free energy [52,56], and this feature naturally caused the *W_a_* value of the PVAc/ITO interface to be high. As for Nylon 66, the *W_a_* value of this polymer seems to be higher than that of the many other polymers that have listed in this work. The reason for this is the high surface polarity (χ_s_ = 16.22%) due to the presence of amide groups instead of aliphatic hydrocarbons in the structure [51,53]. The *W_a_* between PVC and ITO (72.35 ± 2.7 mJ/m^2^) was also found to be higher than that of the many other polymers such as HDPE (63.46 ± 2.5 mJ/m^2^), LDPE (62.56 ± 2.7 mJ/m^2^), PP (63.13 ± 3.3 mJ/m^2^) and SBR (59.45 ± 3.4 mJ/m^2^). Because, replacement of covalent hydrogen atoms by chlorine atoms causing higher adhesional energies and thus higher *W_a_* values, as in the previous determinations of Zisman and coworkers [54,62].

Fluoropolymers are generally known for their nonadhesive behaviours, and they are widely used in many sectors such as aerospace, automotive, electronic, chemical processing, medical devices, and pharmaceutical because of their favourable physical and chemical properties [63]. For this reason, the *W_a_* values between ITO and most common fluoropolymers were also determined in this study. It was observed that significant portion of the fluorinated polymers has lower *W_a_* than that of nonfluorinated polymers due to their low SFE values originating from high electronegativity of fluorine atoms and low polarizability of C-F bonds [55,58,64]. Thus, the *W_a_* values of fluorine containing polymers decreased with the increased of the number of fluorine atoms in the macromolecular structure in the following order: PVF (68.59 ± 2.6 mJ/m^2^) > PVDF (61.65 ± 2.9 mJ/m^2^) > PTrFE (56.03 ± 3.5 mJ/m^2^) > PTFE (44.07 ± 3.7 mJ/m^2^). Also, the *W_a_* value of PPFA (31.26 ± 2.2 mJ/m^2^) was very low when compared to both fluorinated and nonfluorinated polymers. This is because long side chains having CF_2_ units and CF_3_ end groups in perfluorinated acrylate polymers tend to migrate to the outermost part of the polymer surface than being in the bulk [55,58,60,64,65]. Among the fluorinated polymers only PVF shows *W_a_* value that close to commonly used polymers in ITO coatings such as PC, PET, PMMA, while the others are quite far. These results indicate that fluorinated polymers (except PVF) are not suitable for use as substrates in ITO coatings without improving their adhesion properties with appropriate treatments. To sum up, in addition to widely used usual polymers (PC, PET, PMMA), in terms of adhesion strength values PVAc, Nylon 66, PVC, PS, PPS and PVF polymers are promising alternatives that can be used as substrate for ITO coating processes in the future. However, apart from adhesion values, transparency of the aforementioned polymers should also be considered for a successful application.

## 4. Conclusion

The thermodynamic *W_a_* knowledge between coating and substrate is a beneficial parameter that can be used to predict the compatibility of the interlayers in the product to be obtained. In the present study, the compatibility of ITO, which is widely used in optoelectronic industry, with different kind of polymeric substrates was evaluated by utilizing the *W_a_* values of ITO/polymer interfaces. For this purpose, the *W_a_* values between ITO and various polymers were determined by applying the proposals of Dupré, Fowkes and Girifalco-Good to the SFE components obtained by acid-base, geometric mean and harmonic mean approaches. As well as the *W_a_*, the interphase interaction parameters were also determined to comment on the degree of interaction between ITO and polymers. It was found that the *ϕ* values between polymeric surfaces and ITO were close to 1, indicating that all polymers considered in this study have adequate interaction with ITO. According to the calculations made for the determination of *W_a_* values, it has been observed that the magnitude of *W_a_* was related with the total SFE value of the polymeric substrates, and *W_a_* values gradually increased with the increase of the total SFE of the polymer. Calculations performed with acid-base, geometric mean and harmonic mean methods showed similar tendencies in terms of SFE/*W_a_* relationships. Among the considered polymers, PVAC, Nylon 66, PVC, PS, and PPS seems to be a good alternative to PC, PET, and PMMA in terms of *W_a_* values. Among the fluorinated polymers, only PVF has been showed a partially high *W_a_* value with ITO. Overall, there are various alternative polymers that can be considered for use as substrate in ITO coating processes, as well as PC, PET and PMMA, and the interactions of these alternatives with ITO could be controlled by knowing the SFE properties of the polymeric substrate.

## Figures and Tables

**Table 1:** Surface free energy components (mJ/m^2^) of ITO calculated by acid-base (AB), geometric mean (GM) and harmonic mean (HM) approaches.

Method	Substrate	$\gamma_{S}^{LW}\;(\gamma_{S}^{d})$	$\gamma_{S}^{+}$	$\gamma_{S}^{-}$	$\gamma_{S}^{AB}\;(\gamma_{S}^{p})$	$\gamma_{S}^{Tot}$	Reference
AB	PC	31.95	0.00	5.90	0.13	32.08	[21]
	PET	30.07	0.00	5.45	0.00	30.07	[23]
	PET	29.09	0.00	5.15	0.00	29.09	[23]
	Glass	26.60	0.30	7.30	2.80	29.30	[31]
	Glass	26.88	0.00	1.69	0.02	26.90	[4]
	ITO	27.66	0.00	2.11	0.01	27.68	[4]
	Gold	33.26	0.00	6.41	0.05	33.31	[21]
	Silver	25.84	0.00	1.05	0.00	25.84	[4]
	Mean	28.92	0.04	4.38	0.38	29.28	
	Deviation	±2.17	NA	±2.07	NA	±1.91	
GM	PC	31.95	-	-	0.64	32.59	[21]
	PET	27.82	-	-	2.41	30.23	[23]
	PET	27.08	-	-	2.12	29.20	[23]
	Glass	26.88	-	-	0.42	27.30	[4]
	ITO	27.66	-	-	0.36	28.03	[4]
	Gold	33.26	-	-	0.85	34.11	[21]
	Silver	25.84	-	-	0.21	26.05	[4]
	Mean	28.64	-	-	1.00	29.64	
	Deviation	±2.26	-	-	±0.72	±2.28	
HM	PC	33.04	-	-	1.82	34.86	[21]
	Glass	28.66	-	-	2.12	30.78	[4]
	ITO	29.33	-	-	1.98	31.32	[4]
	Gold	34.19	-	-	2.36	36.56	[21]
	Silver	27.77	-	-	1.31	29.08	[4]
	Mean	30.60	-	-	1.92	32.52	
	Deviation	±2.41	-	-	±0.28	±2.55	

NA: not applicable due to very close zero values.

**Table 2:** Water (W), formamide (FA) and diiodomethane (DM) contact angle results of various polymer surfaces.

Polymer	*θ*_W_ (°)	*θ*_FA_ (°)	*θ*_DM_ (°)	Reference
PC^a^	56	32	25	[21]
PVAc	60	43	41	[52]
PC	82	61	32	[21]
Nylon 66	70	50	41	[53]
PPS	96	54	34	[51]
PS	91	74	35	[53]
PVC	87	66	36	[54]
PET	81	61	38	[53]
PMMA	71	59	40	[55]
PVF	80	54	49	[54]
HDPE	102	85	53	[52]
PVDF	82	59	63	[54]
LDPE	102	82	55	[56]
PP	95	77	56	[57]
SBR	98	78	63	[58]
PTrFE	92	76	71	[54]
PTFE	108	92	88	[59]
PPFA	125	109	104	[60]

a shows the contact angle results of O_2_ treated PC.

**Table 3:** Comparison of surface free energy components of various polymer surfaces calculated by acid-base, geometric mean, and harmonic mean approaches.

Polymer	Acid-base approach^a^					GM approach^b^			HM approach^b^		
	$\gamma_{S}^{LW}$	$\gamma_{S}^{+}$	$\gamma_{S}^{-}$	$\gamma_{S}^{AB}$	$\gamma_{S}^{Tot}$	$\gamma_{S}^{d}$	$\gamma_{S}^{p}$	$\gamma_{S}^{Tot}$	$\gamma_{S}^{d}$	$\gamma_{S}^{p}$	$\gamma_{S}^{Tot}$
PC^c^	46.15^d^	0.59^d^	17.54^d^	6.45^d^	52.60^d^	46.15^d^	12.29^d^	58.44^d^	46.25^d^	18.47^d^	64.73^d^
PVAc	39.10^e^	0.55^e^	18.37^e^	6.38^e^	45.49^e^	39.10^e^	12.65^e^	51.76^e^	39.70^f^	17.86^f^	57.56^f^
PC	43.37^d^	0.00^d^	5.70^d^	0.00^d^	43.37^d^	43.37^d^	2.25^d^	45.63^d^	43.63^d^	7.05^d^	50.68^d^
Nylon 66	39.10^e^	0.32^e^	11.05^e^	3.77^e^	42.87^e^	39.10^e^	7.57^e^	46.68^e^	39.70^f^	12.99^f^	52.69^f^
PPS	42.49^e^	0.96^e^	0.00^e^	0.00^e^	42.49^e^	42.49^e^	0.09^e^	42.58^e^	42.80^e^	1.92^e^	44.72^e^
PS	42.03^e^	0.00^e^	4.67^e^	0.00^e^	42.03^e^	42.03^e^	0.59^e^	42.62^e^	42.37^f^	3.74^f^	46.12^f^
PVC	41.56^e^	0.00^e^	4.10^e^	0.00^e^	41.56^e^	41.56^e^	1.32^e^	42.88^e^	41.94^f^	5.31^f^	47.25^f^
PET	40.60^e^	0.00^e^	6.42^e^	0.00^e^	40.60^e^	40.60^e^	2.99^e^	43.59^e^	41.06^f^	7.86^f^	48.92^f^
PMMA	39.61^e^	0.00^e^	15.58^e^	0.00^e^	39.61^e^	39.61^e^	6.98^e^	46.59^e^	40.16^f^	12.43^f^	52.59^f^
PVF	34.83^e^	0.95^e^	4.12^e^	3.95^e^	38.78^e^	34.83^e^	4.51^e^	39.34^e^	35.89^f^	9.21^f^	45.10^f^
HDPE	32.59^e^	0.00^e^	2.04^e^	0.00^e^	32.59^e^	32.59^e^	0.09^e^	32.68^e^	33.93^f^	1.17^f^	35.10^f^
PVDF	26.85^e^	1.80^e^	4.33^e^	5.58^e^	32.43^e^	26.85^e^	5.85^e^	32.70^e^	29.01^f^	9.89^f^	38.90^f^
LDPE	31.45^e^	0.00^e^	1.22^e^	0.00^e^	31.45^e^	31.45^e^	0.14^e^	31.58^e^	32.94^f^	1.33^f^	34.28^f^
PP	30.87^e^	0.00^e^	2.98^e^	0.00^e^	30.87^e^	30.87^e^	1.04^e^	31.92^e^	32.45^f^	3.84^f^	36.29^f^
SBR	26.85^e^	0.01^e^	1.70^e^	0.29^e^	27.14^e^	26.85^e^	1.00^e^	27.85^e^	29.01^f^	3.44^f^	32.45^f^
PTrFE	22.32^e^	0.30^e^	4.18^e^	2.23^e^	24.54^e^	22.32^e^	3.35^e^	25.67^e^	25.18^f^	6.65^f^	31.83^f^
PTFE	13.60^e^	0.29^e^	1.06^e^	1.11^e^	14.72^e^	13.60^e^	1.23^e^	14.84^e^	17.73^f^	2.96^f^	20.69^f^
PPFA	7.30^e^	0.14^e^	0.04^e^	0.15^e^	7.45^e^	7.30^e^	0.17^e^	7.46^e^	11.88^f^	0.07^f^	11.95^f^

a calculated using contact angle values of W-FA-DM liquid triples.

b calculated using contact angle values of W-DM liquid pairs.

c chows the SFE components of O_2_ treated PC.

d taken from reference [21].

e taken from reference [51].

f calculated in this work.

**Table 4:** Interphase interaction parameters (*ϕ*) and work of adhesion (*W*_a_) values (mJ/m^2^) between various polymers and ITO.

Substrate	AB method^a^		GM method^b^		HM method^c^	
	*ϕ*	*W* *_a_*	*ϕ*	*W* *_a_*	*ϕ*	*W* *_a_*
PC^d^	0.971	76.20	0.958	79.72	0.879	80.62
PVAc	0.964	70.37	0.945	74.04	0.879	76.06
PC	0.994	70.83	0.999	73.49	0.960	77.98
Nylon 66	0.983	69.65	0.974	72.43	0.916	75.81
PPS	0.994	70.11	0.990	70.37	0.986	75.21
PS	0.994	69.73	0.998	70.93	0.983	76.15
PVC	0.994	69.34	1.000	71.30	0.975	76.41
PET	0.994	68.53	0.997	71.66	0.957	76.31
PMMA	0.994	67.69	0.977	72.65	0.920	76.12
PVF	0.978	65.93	0.987	67.41	0.946	72.43
HDPE	0.994	61.40	0.991	61.70	0.995	67.27
PVDF	0.952	58.64	0.968	60.30	0.928	66.00
LDPE	0.994	60.32	0.993	60.77	0.997	66.60
PP	0.994	59.76	1.000	61.51	0.991	68.12
SBR	1.000	56.40	1.000	57.46	0.993	64.50
PTrFE	0.982	52.65	0.983	54.23	0.951	61.21
PTFE	0.987	40.96	0.994	41.69	0.955	49.56
PPFA	1.000	29.54	1.000	29.74	0.875	34.51

a SFE components calculated by acid-base approach were used to determine *ϕ* and *W_a_* values.

b SFE components calculated by geometric mean approach were used to determine *ϕ* and *W_a_* values.

c SFE components calculated by harmonic mean approach were used to determine *ϕ* and *W_a_* values.

d shows the *ϕ* and *W_a_* values between O_2_ treated PC and ITO.
